# Real-time monitoring strategies for optimization of *in vitro* transcription and quality control of RNA

**DOI:** 10.3389/fmolb.2023.1229246

**Published:** 2023-09-11

**Authors:** Kyung Hyun Lee, Jaehwi Song, Seongcheol Kim, Seung Ryul Han, Seong-Wook Lee

**Affiliations:** ^1^ R&D Center, Rznomics Inc., Seongnam, Republic of Korea; ^2^ Department of Bioconvergence Engineering, Research Institute of Advanced Omics, Dankook University, Yongin, Republic of Korea

**Keywords:** *in vitro* transcription, IVT, transcription, real-time monitoring, RNA

## Abstract

RNA-based therapeutics and vaccines are opening up new avenues for modern medicine. To produce these useful RNA-based reagents, *in vitro* transcription (IVT) is an important reaction that primarily determines the yield and quality of the product. Therefore, IVT condition should be well optimized to achieve high yield and purity of transcribed RNAs. To this end, real-time monitoring of RNA production during IVT, which allows for fine tuning of the condition, would be required. Currently, light-up RNA aptamer and fluorescent dye pairs are considered as useful strategies to monitor IVT in real time. Fluorophore-labeled antisense probe-based methods can also be used for real-time IVT monitoring. In addition, a high-performance liquid chromatography (HPLC)-based method that can monitor IVT reagent consumption has been developed as a powerful tool to monitor IVT reaction in near real-time. This mini-review briefly introduces some strategies and examples for real-time IVT monitoring and discusses pros and cons of IVT monitoring methods.

## Introduction

Since the global COVID-19 pandemic, RNA vaccines have been hailed as new vaccines to quickly respond to infectious diseases (Dolgin, 2021). Furthermore, RNA is now considered as a feasible modality for therapeutic applications as well as vaccine application (Huang et al., 2022). In addition to linear RNA modality, circular RNA (circRNA) (Liu and Chen, 2022) and self-replicating RNA (Dailey et al., 2022) are also considered as promising RNA modalities.

For efficient translation and *in vivo* stability of linear mRNA vaccine as an example, 5′-capping (Ramanathan et al., 2016) and poly(A) tail (Eckmann et al., 2011) are required for linear mRNA. 5′-capping reaction can be accomplished during *in vitro* transcription (IVT) (Contreras et al., 1982) by co-transcriptional capping using dinucleotide such as Anti-Reverse Cap Analog (ARCA) system (Peng et al., 2002) or after IVT through additional enzymatic reaction using an enzyme such as vaccinia capping enzyme and related reagents for cap-0 (Shuman, 1990) and cap 2′-O-methyltransferase for cap-1 and cap-2 structures at the 5′end of the RNA with a cap-0 as substrate (Lockless et al., 1998). Poly(A) tail can be directly transcribed from DNA template or can be added by enzymatic reaction after IVT using poly(A) polymerase such as *E. coli* poly(A) polymerase (Cao and Sarkar, 1992). IVT reagents such as NTPs and Mg^2+^ are also important for IVT efficiency and product yield (Pregeljc et al., 2023). In addition, modified nucleotides such as m1ψ (*N1*-methylpseudouridine-5′-triphosphate) are required for linear mRNAs to have efficient translation (Svltkin et al., 2017) and avoid unwanted innate immune responses (Mu and Hur, 2021) for *in vivo* applications.

As an example of other RNA modalities, circRNA also can be generated by IVT (Petkovic and Müller, 2015; Lee et al., 2022a) using group I intron ribozyme-based strategy such as permuted intron-exon method (Puttaraju and Been, 1992). In contrast, chemical- or ligase-based circRNA preparation methods require an additional step after precursor RNA preparation by IVT (Lee et al., 2022a). For circRNA in general, 5′-capping is not necessary for translation and poly(A) is optional to improve expression mediated by internal ribosome entry site (IRES) possibly through polyadenylate binding proteins (Wesselhoeft et al., 2018). Interestingly, nucleotide modifications are not compulsory for circRNA to avoid unwanted innate immunity (Wesselhoeft et al., 2018), and this observation needs to be demonstrated by clinical trials in the near future.

Therefore, IVT process would be fundamental for determining the yield and purity of RNA products. To produce large amounts of RNA-based vaccines and therapeutics with high purities, useful IVT monitoring methods are needed to understand molecular mechanisms and to monitor target RNA production, efficacy of the aforementioned enzymatic reactions, and reagent consumption.

Traditionally, oligonucleotides and polynucleotides including RNAs have been routinely analyzed by various analytical techniques such as polyacrylamide gel electrophoresis (PAGE, Loening, 1967), agarose gel electrophoresis (Koontz, 2013), capillary electrophoresis (Wei et al., 2022), and high-performance liquid chromatography (HPLC) (Thayer et al., 1996). However, those methods are neither real-time or near real-time methods for IVT monitoring. They are generally used for endpoint analysis. Real-time IVT monitoring methods would be ideal to better understand the IVT reaction progress and/or to adjust the IVT condition immediately during IVT process. Here, we briefly review reported real-time or near real-time IVT monitoring methods and discuss their strengths and weaknesses.

### Real-time IVT monitoring strategies using light-up RNA aptamer and fluorescence dye pairs

**FIGURE 1:**
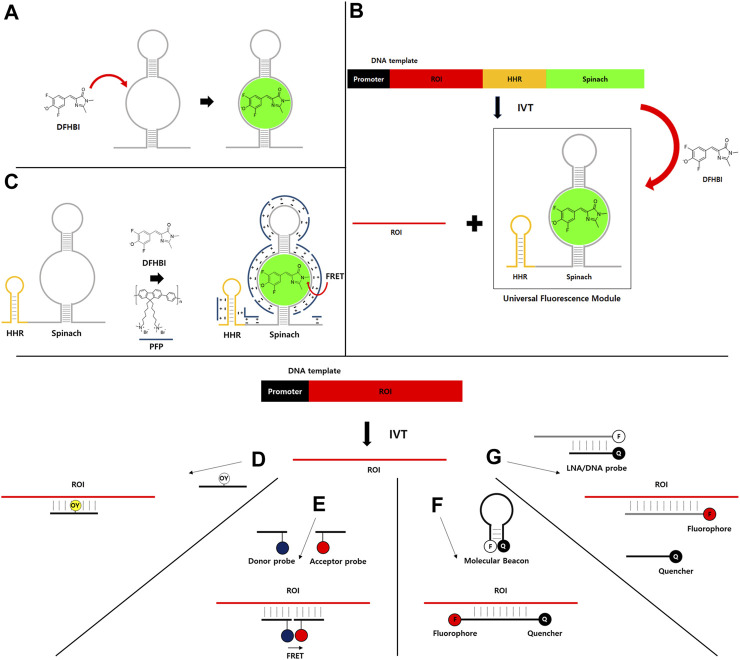
**(A–C)** Light-up RNA aptamer and fluorescence dye pair for real-time IVT monitoring. **(A)** An example of light-up RNA aptamer (Spinach) and fluorescence dye (DFHBI) pair. DFHBI dye is nonfluorescent due to subtle movement. However, its nonfluorescent form can switch to a fluorescent form when it binds to a specific RNA aptamer such as Spinach. **(B)** DNA template containing promoter and a universal fluorescence module (UFM) consisting of a highly active hammerhead ribozyme (HHR) and Spinach RNA aptamer can be used to monitor the synthesis of RNA of interest (ROI) during IVT in the presence of DFHBI dye. **(C)** FRET between water-soluble conjugated polymer (PFP, blue line) that binds to RNA by nonspecific electrostatic interaction as a FRET donor and RNA aptamer/DFHBI complex as a FRET acceptor. **(D–G)** Fluorophore-labeled antisense probe-based strategies for real-time IVT monitoring. **(D)** Fluorophore-labeled antisense probe for fluorescence turn-on. **(E)** FRET-based strategy. **(F)** Molecular beacon-based strategy. **(G)** Strategy using fluorophore-labeled and quencher-labeled probes.

In nature, there are no green fluorescent protein (GFP)-like RNAs to visualize RNA by tagging. Therefore, RNA aptamers that can bind and turn on nonfluorescent fluorogens have been developed for RNA imaging and/or detection *in vitro* (Babendure et al., 2003), in bacteria (Lee et al., 2010), and in mammalian cells (Paige et al., 2011; Swetha et al., 2020; Figure 1A). Such light-up RNA aptamers can be selected by SELEX (Systematic Evolution of Ligands by EXponential enrichment) method (Ellington and Szostak, 1990; Tuerk and Gold, 1990), which can specifically bind to the target molecule such as light-up fluorophores. Therefore, the selected light-up RNA aptamer that is genetically encodable can be used for tagging of RNA of interest.

Generally, nonfluorescent dye for the light-up aptamer strategy has extremely low quantum yield due to vibrational de-excitation (Oster and Nishijima, 1956). However, RNA aptamer can bind and stabilize the nonfluorescent dye in a more fluorescent conformation by restricting the vibration, resulting in fluorescence light-up (Babendure et al., 2003). Therefore, RNA aptamer can be tagged to RNA of interest (ROI) like GFP tagging strategy. It is then incubated with a light-up fluorescence dye for the detection and/or visualization of ROI similar to the detection of protein of interest by fluorescence intensity of GFP.

Among various light-up aptamer and fluorescence dye pairs developed so far, Spinach (Paige et al., 2011) and related RNA aptamers such as Broccoli (Filonov et al., 2014), Mango (Dolgosheina et al., 2014), Corn (Song et al., 2017), and Pepper (Chen et al., 2019) are being widely used by researchers (Swetha et al., 2020). Some light-up fluorescence dyes such as DFHBI (Paige et al., 2011), DFHBI-1T (Song et al., 2014), and DFHO (Song et al., 2017) are commercially available.

For the application of light-up RNA aptamer strategy, the signal from the real-time IVT monitoring method should be independent from the polymerase, ROI, and other components to accurately monitor the transcription activity. To this end, a simple, robust, and universal method has been developed to analyze the quality and quantity of transcribed RNA during IVT (Höfer et al., 2013). In this strategy, Spinach RNA aptamer and DFHBI dye are used in a high-throughput manner (Figure 1A). A universal fluorescence module (UFM) consisting of a highly active hammerhead ribozyme (HHR) and light-up RNA aptamer is independent of ROI sequences. Thus, UFM can be used as a universal platform for real-time IVT monitoring of any ROI. As HHR is introduced behind the ROI to cleave the RNA aptamer part from the ROI, UFM can be released during IVT. Released RNA aptamer parts and fluorescence dyes can be efficiently purified out by the purification method such as HPLC as the sizes will be generally much smaller than ROI. Therefore, only ROI can be obtained as the final product without extraneous sequences for further applications (Figure 1B). As HHR generates a homogenous 3′end by removing the aptamer portion comprising of heterogenous 3′ends generated by the addition of untemplated nucleotide by T7 polymerase activity (Akoopie and Müller, 2018), poly(A) tailing reaction with the purified IVT RNA would provide a narrower distribution of poly(A) compared to reactions with RNAs comprising of heterogenous 3′ends.

Detection speed with real-time IVT monitoring using light-up aptamer and fluorescent dye pair is at least 100 times faster than conventional polyacrylamide gel electrophoresis (PAGE) method (Valentini et al., 2022), whereas PAGE method shows RNA bands that can provide purity information of RNA transcripts. Although the light-up aptamer-based method cannot provide quality information of IVT, eventually the purity of RNA products can be checked during compulsory HPLC purification process (Karikó et al., 2011) and/or by other endpoint analytical methods for applications in cells and *in vivo*.

If transcribed RNAs will not be used for further applications after real-time IVT monitoring using light-up aptamer and dye pair, in other words, if IVT is only monitored to check transcription activity itself for IVT optimization, introduction of ribozyme domain would be unnecessary (Valentini et al., 2022). For example, effects of IVT components have been investigated using Broccoli aptamer-based (Kartje et al., 2021) or iSpinach aptamer (Autour et al., 2016)-based monitoring of transcription activity in a real-time (STAR) system (Valentini et al., 2022). An approach based on contact quenching of fluorophores linked to quenchers such as dinitroaniline and fluorophore-binding aptamer has also been used for real-time IVT monitoring (Sunbul and Jäschke, 2013). Similarly, the fluorophore-quencher conjugate is not fluorescent owing to contact quenching. It becomes fluorescent upon binding to the tagged RNA aptamer on the target RNA during IVT.

Fluorescence resonance energy transfer (FRET)-based monitoring strategy using UFM and water-soluble conjugated polymer has also been reported (Li et al., 2019; Figure 1C). Briefly, poly (9, 9-bis (6′-N, N, N,-trimethylammonium) hexyl) fluorene-co-alt-1,4-phenylene) bromide (PFP) is used as a FRET donor for DFHBI dye which could bind to RNA aptamer as a FRET acceptor. Strong electrostatic interaction between PFP and RNA aptamer/DFHBI complex allows FRET. The method can amplify fluorescence signals to improve detection sensitivity compared to a UFM strategy with intensity-based fluorescence detection. Therefore, the limit of detection (LOD) is near 10-fold lower (LOD = 0.29 nM) than that of UFM alone (LOD = 2.8 nM) as PFP does not interfere with IVT and the variation of background signal is greatly reduced by FRET measurement.

However, one concern would be fast fluorescence decay by photoconversion after illumination (Han et al., 2013; Lee et al., 2022b) during real-time monitoring which requires continuous illumination of the fluorescence dye. To resolve this issue, unnatural base pair technology has been employed for real-time IVT monitoring (Lee et al., 2022b). In that technology, DFHBI-conjugated unnatural nucleotide is incorporated into specific position in Spinach RNA during IVT for fluorescence light-up, resulting in improved photostability, thermal stability, and ion sensitivity. However, that method can only be used for IVT monitoring and optimization due to incorporated unnatural nucleotide that could be inappropriate for downstream applications, although it is much more robust for IVT monitoring than the conventional method. In addition, it is possible that RNA aptamer with stable hairpin loop structure may slow down or terminate the T7 RNA polymerase activity.

### Real-time IVT monitoring strategies using fluorophore-labeled antisense probes

Various other strategies have also been employed to monitor IVT in real time. For example, a simple strategy by directly incorporating γ-fluorophore-labeled UTP into RNA during transcription has been used to monitor IVT (Dunkak et al., 1996). However, produced RNAs with dye modification are inappropriate for downstream applications.

Instead of directly incorporating the fluorophore into RNA target, fluorophore-labeled antisense probes such as a fluorescent DNA probe that can enhance fluorescence on hybridizing with a target RNA have been developed for real-time IVT monitoring. For example, oxazole yellow (YO) is normally non-fluorescent (Ishiguro et al., 1996). However, the dye shows enhanced fluorescence by intercalation into double-stranded DNA (dsDNA). YO-linked complementary oligonucleotide can enhance the fluorescence in the presence of target RNA products in IVT reaction (Figure 1D). In addition, fluorescence correlation spectroscopy (FCS) strategy can be used for IVT monitoring using a fluorophore-labeled probe (Nomura and Kinjo, 2004). FCS can sensitively measure fluctuations in fluorescence intensity of the fluorescent molecule in solution, which is dependent on molecular weight and concentration (Maiti et al., 1997). Thus, fluorescence correlation functions were analyzed using a simple two-component model with a fast-moving component of free probe and a slow-moving component of the probe-RNA hybrid as an RNA product.

Two fluorescent DNA probes can be used for FRET-based IVT monitoring (Figure 1E). Two 15-mer DNAs with one labeled with Bodipy493/503 as the FRET donor and the other labeled with Cy5 as the acceptor have been used to generate a FRET signal when hybridized to adjacent sites of the target RNA (Sei-lida et al., 2000). In this strategy, probes are hybridized to synthesizing RNAs before they are folded to form secondary structures. Thus, there is no need to select efficient binding sites on the target RNA for probes. In another study using Cy3 as FRET donor and Cy5 as acceptor (Niederholtmeyer et al., 2013), mRNA secondary structure and target site location are important parameters for efficient FRET (Niederholtmeyer et al., 2013). In that study, a target site located in the 3′untranslated region (UTR) was sensitive for real-time quantitation of the produced mRNA (LOD = 50 nM) by designing to reduce secondary structure. Recently, a binary fluorescence quencher (BFQ) probe consisting of two single-stranded DNA (ssDNA) probes that were modified by a donor fluorophore and a quencher fluorophore and the PBCV-1 DNA ligase were used for FRET-based real-time quantitative *in vitro* transcription assay (Zhang et al., 2023). The PBCV-1 DNA ligase ligates the two ssDNA probes hybridized to the RNA target at adjacent regions into one single DNA strand to stabilize the FRET signal. Therefore, the amount of fluorescence quenching that is proportional to the produced RNA amounts will be detected during IVT.

A simple molecular beacon (MB) strategy using a DNA probe containing a fluorophore and quencher pair has been applied for real-time IVT monitoring (Liu et al., 2002; Figure 1F). MB probe is structured as target-specific antisense oligonucleotide containing a proximate fluorophore and quencher pair. Upon binding to its target RNA during IVT, the probe undergoes a structural rearrangement, resulting in fluorescence recovery by separating the proximate fluorophore and quencher pair. 2′-*O*-methylribonucleotide backbone can be used for MB probe to increase the specificity by eliminating the background fluorescence that might be generated due to formation of duplex RNA between MB probe and its complementary RNA synthesized nonspecifically by RNA polymerase in a promoter-independent manner (Marras et al., 2004). The MB method is simple and easy by using cheap commercial oligonucleotides synthesis service for preparing fluorophore- and quencher-modified probes. However, if IVT products should be purified, how to separate antisense probes with highly complementary sequences to the target RNA should be considered.

A locked nucleic acid (LNA) (Veedu and Wengel, 2010) probe and quencher oligonucleotide pair has been used for real-time monitoring of transcription in a HeLa-based mammalian cell-free expression system (Wang et al., 2018). In that study, 21 nucleotide-long LNA probe consisting of alternating LNA/DNA monomers with Texas Red at the 5′-end and 10 nucleotide-long LNA/DNA oligonucleotides labeled with Iowa Black RQ as a quencher on the 3′-end complementary to the 5′-end of LNA probe have been designed for a double-stranded LNA probe (Figure 1G). Once the LNA probe binds to the target RNA during transcription, the binding is stabilized, preventing recombining with the quencher strand and resulting in recovery of fluorescence intensity (LOD = 2 nM).

### HPLC-based IVT monitoring strategy

HPLC is a powerful and sensitive analytical method that can monitor various reactions and provide qualitative and quantitative information for various materials including nucleic acids (Thayer et al., 1996). HPLC is also an important purification method for obtaining pure RNA products to eliminate contaminants which can induce unwanted innate immune responses (Karikó et al., 2011).

**FIGURE 2:**
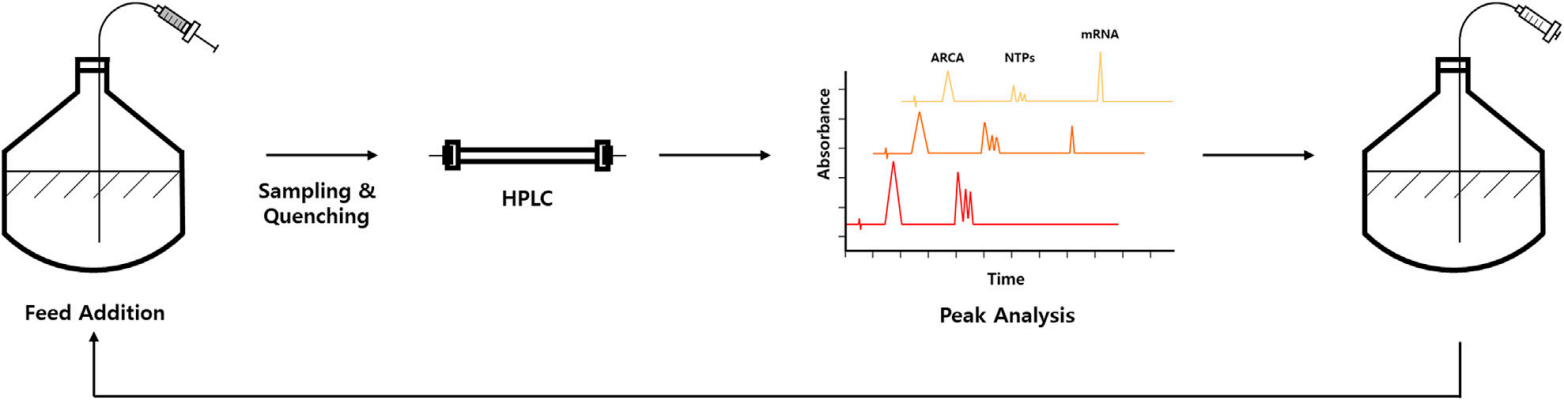
Schematic representation of at-line HPLC-based IVT optimization workflow, which allows fed-batch approach. Representative chromatograms are shown in peak analysis part. Elution peaks corresponding to anti-reverse cap analog (ARCA), NTPs, and mRNA are indicated in chromatograms.

Recently, at-line HPLC strategy has been reported for IVT monitoring at a specific time point (Pregeljc et al., 2023). In this strategy, IVT sample is quenched for injection for HPLC analysis at a specific time point (Figure 2). Generally, run-time of traditional reverse-phase HPLC methods that can also resolve IVT components such as NTPs is too long (20–30 min). To overcome this issue, Pregeljc et al. have developed a high-throughput HPLC assay for simultaneous quantification of IVT components such as NTPs, plasmid, capping agent, and RNA (Korenč et al., 2021) and applied this method to follow IVT reactions at-line with a minimal lag (Pregeljc et al., 2023).

By using at-line HPLC method, IVT reaction conditions (e.g., Mg^2+^, plasmid, and NTP concentrations) can be optimized by monitoring NTP consumption and RNA production in near real-time. More interestingly, a fed-batch approach for IVT that can extend time and yields by continuously adding IVT components to the reaction mixture for therapeutically relevant mRNA has been demonstrated (Pregeljc et al., 2023; Figure 2). Quantification of mRNA is also coupled with exonuclease digestion to monitor capping efficiency of the produced mRNA. Briefly, 5′polyphosphatase is used to remove phosphates from 5′-triphosphate of uncapped RNA. In contrast, the capped RNA is resistant to this enzyme. Next, Terminator™ 5′-phosphate-dependent exonuclease was treated to digest 5′-monophosphorylated RNA. Therefore, the capping efficiency can be calculated by comparing with control sample without exonuclease digestion (Chiron and Jais, 2017; Pregeljc et al., 2023).

As for future directions, Pregeljc et al. foresee a coupling of at-line HPLC-based IVT monitoring strategy with multi-parallel automated bioreactor systems, enabling controlled and continuous addition of IVT components such as NTP and Mg^2+^ by monitoring consumption kinetics with control of various parameters. In addition, quantitative information obtained by HPLC-based IVT monitoring could reduce uncertainty in RNA quantification before downstream purification process (Pregeljc et al., 2023).

### Spectroscopic methods to improve IVT monitoring strategy

Spectroscopic methods also would be applied to strengthen the real-time IVT monitoring methods in the near future. For examples, raman spectroscopy is likely to be applied for RNA manufacturing also by measuring NTP consumption (Matuszczyk et al., 2023). Circular dichroism (CD) that sensitively probes the chirality of nucleic acids would be useful to analyze nucleotide modifications and functional structure of RNA (Valentini et al., 2022) during IVT and the database is currently available as a public repository (Zhang et al., 2023). Time-resolved infrared spectroscopy would also be an option to analyze RNA folding that affects the function of RNA such as aptamer (Brauns and Dyer, 2005).

## Conclusion

In this mini review, we briefly introduced real-time IVT monitoring strategies. Among various strategies briefly explained above, light-up aptamer-based method is widely being used for RNA detection and various applications (Swetha et al., 2020). At-line HPLC-based strategy has been recently reported as a powerful tool for IVT monitoring, which can even monitor IVT reagents’ consumption and allow fed-batch approach (Pregeljc et al., 2023). Although this method is near real-time rather than real-time, the strength of the HPLC-based strategy is that all components of the IVT reaction can be monitored, whereas other methods can only monitor the final RNA product. Spectroscopic methods would be also helpful to strengthen the real-time IVT monitoring strategies.

Each method has its own pros and cons for IVT monitoring. Researchers need to wisely choose an appropriate method for their research purposes. Real-time IVT monitoring would be more useful for RNA manufacturing by IVT if fluorescence-based strategies and HPLC-based methods are used complementarily. As research on RNA therapeutic and RNA vaccine is receiving a lot of attention, real-time IVT monitoring methods are expected to be rapidly advanced.
